# Crista Galli Pneumatization Complicating Olfactory Groove Meningioma Resection: A Case Report

**DOI:** 10.7759/cureus.101289

**Published:** 2026-01-11

**Authors:** Vasileios Kavouridis, Ekkehard M Kasper

**Affiliations:** 1 Neurological Surgery, Aristotle University of Thessaloniki, Thessaloniki, GRC; 2 Neurological Surgery, Boston Medical Center - Brighton, Boston, USA

**Keywords:** case report, crista galli pneumatization, meningioma, olfactory groove, skull base

## Abstract

Pneumatization of the crista galli (PCG) is an anatomic variant of the osseous skull base. Olfactory groove meningiomas (OGMs) arise in the vicinity of the crista galli, and PCG may be an obstacle to complete resection from a preferred unilateral approach.

A 22-year-old male presented with symptoms of chronic frontal headaches and was found to harbor a 4cm OGM. Preoperative imaging workup also revealed the presence of a very large PCG with a height of 27mm above the floor of the anterior fossa.

Given the presence of PCG, we elected to adjust our conventional approach of a unilateral pterional/subfrontal craniotomy to a modified bilateral frontal craniotomy, which was a combination of a large right-sided pterional and a small left-sided frontolateral osteotomy, sparing the midline. This allowed excellent access to the floor of the anterior fossa, allowing us to achieve a favorable Simpson 2 resection. Apart from iatrogenic anosmia, the patient had an uneventful recovery.

The preoperative work-up of OGM patients should include dedicated CT imaging of the skull base to assess potential variations in the aeration status of the paranasal sinuses and ethmoidal cells. This is of great clinical relevance as it allows for the detection of variations such as a PCG. This can prompt an adjustment of the operative strategy to minimize the risk of postoperative cerebrospinal fluid (CSF) leak and meningitis.

## Introduction

Anatomic variations of the paranasal sinuses and skull base air pockets are commonly described in the literature [1,2]. Among these, some form of pneumatization of the crista galli (PCG) occurs with an estimated incidence of 16% in the general population. PCG derives its aeration from either the frontal sinus or the ethmoidal cells [3,4].

Among the anterior cranial fossa skull base tumors, olfactory groove meningiomas (OGMs) arise in the immediate vicinity of the crista galli (CG) and cribriform plate. For that reason, anatomical variations in the configuration of these structures can have a significant influence on surgical strategy and execution. More specifically, the presence of a PCG can complicate attempts to achieve a desirable complete (Simpson grade 1) resection and can furthermore be associated with significant morbidity since drilling of an undetected PCG can incur a cerebrospinal fluid (CSF) leak with risk for postoperative meningitis. Therefore, preoperative workup of these tumors should include comprehensive imaging with both a high-resolution contrasted MR as well as a thin-cut CT with bone windows in three planes of the skull base. The latter aids in surgical planning as meticulous attention can be paid to aeration patterns and structural variations of the paranasal sinus.

Herein, we present a case of an OGM resection in the setting of an unusually large CG. The preoperative detection of the aerated skull base structure altered our preferred standard unilateral surgical resection plan. In order not to violate the CG, we opted for an individualized modified bilateral surgical approach.

## Case presentation

The patient was a 22-year-old male student, without significant past medical history, who originally presented to the emergency department complaining of headaches of 10 days' duration. He described these headaches as constant, frontotemporal, “vice grip”-like, interfering with sleep. The patient also reported nausea and neck muscle stiffness, with severe pain upon sneezing. He denied blurry vision, anosmia, seizures, or any other focal neurologic deficit. In retrospect, he had had episodic and occasional headaches over the course of his life, sometimes with migraine-like symptoms, but only lately had these become more intense and persistent. 

A head CT was initially performed and demonstrated a 4cm predominantly right-sided mass with transfalcine extension towards the left, originating from the anterior skull base. There was a local mass effect on the frontal poles with surrounding parenchymal edema, both vasogenic and from venous outflow obstruction. Most importantly, the scan was also remarkable for a high-riding, large CG with significant pneumatization. The air cell inside the crista clearly showed a communicating channel with the air-filled left frontal sinus. 

**Figure 1:**
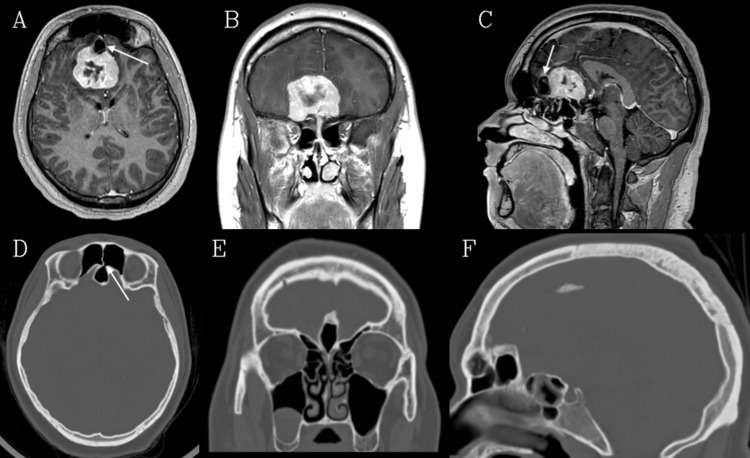
Preoperative imaging A, B, C: Axial, coronal, and sagittal T1 contrasted images, respectively, showing the extent of the OGM. The aerated CG is also visible on the axial and sagittal images, sitting against the anterior border of the tumor (arrows). D, E, F: Axial, coronal, and sagittal CT images (bone window), respectively, demonstrating the characteristics of the CG. Panel D shows how it derives its aeration from the left frontal sinus (arrow). It can be classified as type I, given its growth entirely above the cribriform plate.

A brain MRI more clearly delineated the nodular tumor as a 3.9 x 4 x 3.3cm enhancing lesion in the anterior skull base with significant perilesional edema and mass effect onto the corpus callosum and frontal horns of the lateral ventricles (Figure 1). CTA of the head was undertaken and was notable for vascular feeders coming from the ethmoidal arteries. There was a mass effect on bilateral anterior cerebral arteries (ACAs), which were posteriorly displaced. 

The patient was admitted to the neurosurgery service for further work-up and treatment. He was started on steroids and antiepileptic drugs for prophylaxis. Given the size of the lesion, it was decided to proceed with a formal pre-operative angiography to possibly pursue presurgical embolization. The digital subtraction angiograph (DSA) was completed the day before surgery, with the key observation that the tumor blood supply was primarily arising from the left ophthalmic artery, preventing takedown of the feeders from this side. There were also contributions from the right distal internal maxillary artery as well as the right ophthalmic artery. Due to the complex vascular pattern, only minimal embolization could be performed, and he was then taken to the operating room (OR) the following day for definitive surgery. 

Standard neuronavigation was used to delineate landmarks such as the superior sagittal sinus and bilateral frontal sinus and to map out the optimal trajectory. Instead of the most commonly chosen right-sided pterional/frontolateral approach, we decided to tailor our access craniotomies and employ a bilateral approach via a coronal incision. We first harvested a pericranial flap. Aiming to avoid the osseous midline structures, and in an effort not take down the PCG, we created two separate bone flaps (right larger than left). The craniotomy was performed in conventional steps on each side and included drilling of some of the orbital roof and shaving off some of the interdigitations. We then proceeded with a right-sided wide dural opening for CSF release from the lateral and basal cisterns. Once brain relaxation was accomplished (aided by hyperventilation, furosemide, and mannitol), we undertook the next step of tumor devascularization: taking down the anterior ethmoidal feeders on each side above the cribriform plate. This was followed by piecemeal debulking and careful dissection of the posterior borders. 

**Figure 2:**
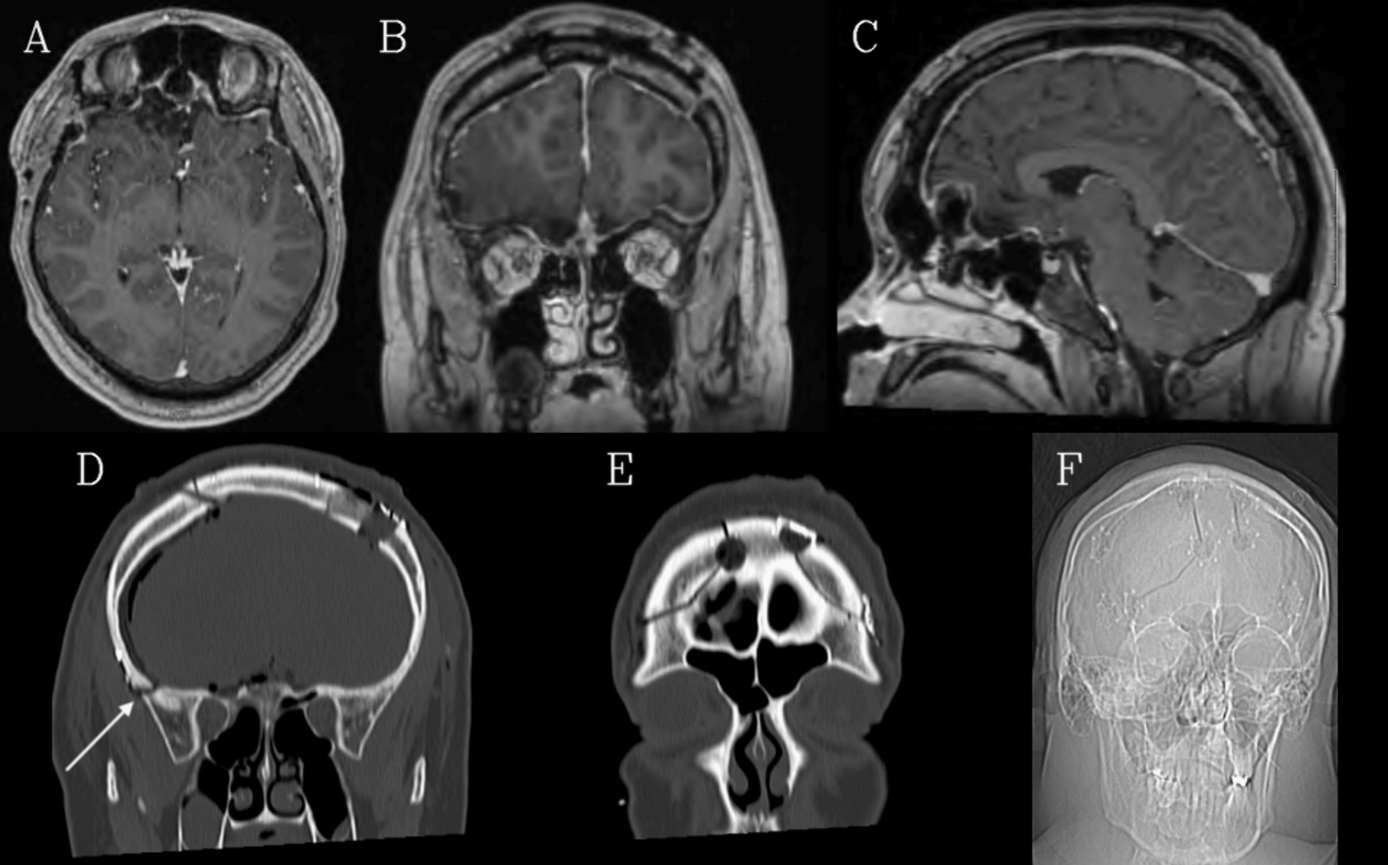
Postoperative imaging A, B, C: Axial, coronal, and sagittal T1 contrasted images, respectively, demonstrate near-total resection of the mass. D, E, F: Coronal CT (bone window and accompanying scout image) showing the outlines of bilateral frontal craniotomies, with the right-sided one extending more inferiorly towards the skull base, given that the mass was predominantly on the right (arrow).

On the left side, one larger distal branch of the frontopolar artery supplying the capsule of the tumor had to be sacrificed, and a Weck-clip was applied to the vascular stump. At the end of the case, we had achieved a Simpson grade 2 resection with minimal microscopic dural infiltrate left behind, which was addressed with cauterization. A small muscle + gel-foam + hydrogel sandwich was used to prevent any possible CSF leak through the devascularized cribriform plate. Histological examination demonstrated a WHO grade 1 meningioma, though it displayed an increased proliferative index (MIB-1) of 18%; next-generation sequencing was negative for aggressive genotype. Early postoperative MRI showed minimal residual dural abnormalities in the left subfrontal area without other concerning features (Figure 2). The patient complained of anosmia postoperatively, but his course was otherwise unremarkable, and he was discharged on postoperative day two. Given the high mitotic activity, the tumor board recommended postoperative adjuvant radiation, and he underwent conventional external beam 3D-conformal intensity-modulated radiation therapy (IMRT) to the surgical bed postoperatively. He was last seen in follow-up two years after surgery with a stable clinical status and no residual or recurrent disease on imaging (Table 1). 

**Table 1:** Summary of preoperative, intraoperative, and postoperative data CT: computed tomography; PCG: pneumatized crista galli; MRI: magnetic resonance imaging; DSA: digital subtraction angiography.

Category	Details
Patient Demographics	22-year-old male
Presentation	Subacute worsening of chronic headaches, nausea, and neck stiffness
Preoperative Imaging	CT: 4 cm anterior skull base mass, large PCG communicating with the left frontal sinus. MRI: 3.9 x 4 x 3.3 cm enhancing olfactory groove meningioma. DSA: Supply from bilateral ophthalmic & right internal maxillary arteries; minimal embolization possible.
Key Anatomic Variant	Large (28x17x18 mm), Type I PCG, aerated from the left frontal sinus.
Surgical Strategy	Modified bilateral frontal craniotomy (right > left) tailored via neuronavigation to preserve PCG.
Resection Outcome	Simpson Grade 2 (minimal dural residue, cauterized).
Complication	Postoperative anosmia.
Adjuvant Therapy	Fractionated radiotherapy (IMRT) due to high MIB-1 index (18%).
Follow-up	No recurrence at 2 years.

## Discussion

The CG is a bony eminence that protrudes atop the cribriform plate in the midline. Its posterior edge serves as the anterior attachment for the falx cerebri. From an embryological standpoint, this portion of the viscerocranium is derived from the meso-ethmoidal cartilage. Ossification starts at about two to three months of fetal age and appears to be completed in most individuals at around 24 months [5,6]. There is a remarkable variation in the extent and anatomical configuration of the paranasal sinuses, including their aeration pattern [1,7]. Secondary aeration of the bone helps to decrease the weight of the compact bones, and pneumatization of the skull base with extension into the CG is not an uncommon occurrence with an incidence in the literature between 2.5-37% (median 16.5%) [2,8-15]. Given its embryological origin from the ethmoid bone, it was traditionally thought that aeration of the CG would arise from the ethmoid complex. Recent work has, however, challenged this notion. Som et al. [4] examined CT scans of 200 adults and concluded that PCG came in most individuals exclusively from one of the frontal sinuses and not from displaced ethmoid cells. Supportive of their theory was the fact that PCG incidence was very low at ages less than seven years, when the frontal sinus is still rudimentary. Subsequently, increases in numbers are found in parallel to the respective age and extent of sinus development, an observation shared also by Tetiker et al. [16].

Hajiioannou classified the configuration of the CG into three anatomic subgroups with regard to extension above or below the cribriform plate, with group I being entirely above it and group III having >50% of the CG height below the plate [17]. Interestingly, these authors found increasing pneumatization rates in the subgroups II-III compared to group I. Kim et al. made a similar observation, with almost all their cases of PCG deriving from the frontal sinus. However, they did not find a statistically significant correlation between pCG rate and anatomical CG type [3].

In our patient, the observed CG was quite sizeable (dimensions of 28x17x18mm). Upon review of the skull base CT, it was evident that the pneumatization channel occurred as an extension of the left frontal sinus, and its pneumatization pattern could be clearly classified as type I, i.e., with the PCG exclusively localized above the level of the cribriform plate. 

While there is adequate literature on the incidence, morphometrics, and putative origin of PCG, there is a dearth of publications focusing on the significance of this variation on operative outcome. We feel that this is an understated problem, as it profoundly influences the management of anterior skull base lesions in general and olfactory groove meningiomas in particular. Unfortunately, to the best of our knowledge, this has not been addressed in any of the pertinent reports on OGM published thus far. 

In one of the few other available related neuro-oncology studies, Akiyama et al. [18] examined imaging of 300 patients with brain tumors, of which 24 were operated on for skull base masses with a bilateral interhemispheric approach. These authors reported a PCG incidence of 9.3%; however, none of the reported surgical skull base cases displayed this variation. The CG was drilled away in the vast majority of the subgroup of their study patients (22/24), and it was remarked that residual CG height after intraoperative drilling was 7.2mm. The only conclusion the authors provided related to this observation was that, in their opinion, if CG height is >7.2mm, there is a high probability that it will need to be partially removed. In the presence of PCG, they urged particular attention to this feature to avoid a postoperative CSF leak, especially if such aeration were to arise from the ethmoids, which would then require intradural skull base closure. 

This differs from the observation made in our case. Here, and based entirely on diligent presurgical CT review, we elected to avoid a unilateral approach necessitating removal of the CG altogether. Instead, we opted for a rather simple, yet superior exposure afforded by an image-guided tailored bifrontal craniotomy that spared the frontal sinus, which provided us with a favorable angle of attack to both sides of the tumor. The right side is for bulk resection, and the left for removal of the tumor segment otherwise hidden behind the CG when seen from a unilateral access route. This adaptation of the surgical strategy made a successful gross total resection possible without any need to drill down the PCG - thus avoiding the risks of CSF leak altogether.

Another aspect that should be emphasized as a valuable decision-making tool in OGM surgery is the use of neuronavigation during the planning phase. Certainly, an experienced surgeon does not necessarily need an imaging platform to localize these formidable lesions in the anterior cranial fossa. However, despite not being standard practice, it can be very advantageous to employ this tool from more than one perspective. An obvious benefit is in mapping the frontal sinus and avoiding its inadvertent entry, especially in cases where a large frontal and medial component of the craniotomy is decided. In this setting, strategic use of image guidance, employing thin-cut bone windows, can help to plan a trajectory that optimizes viewing angles. Once intracranially, the soft tissue MRI sequences will allow to minimize tissue disruption while maintaining optimal overview of the anatomy of the tumor circumference (e.g., ACA vasculature frequently draped over the posterior-superior aspect of the mass), thus leading to a more individualized operative approach. This is especially important in large OGM surgery, given that no single surgical approach has emerged as the gold standard over the years. 

Recent studies of complications related to OGM surgery favor unilateral approaches for resection to minimize complications. In that spirit, Downes et al. [19] described a four-stage approach for resection of giant OGMs that employed neuronavigation to fashion a tailored unilateral fronto-orbital craniotomy, thus avoiding potential complications of bifrontal or pterional approaches. 

We routinely employ neuronavigation for all of our tumor cases, and this was true for the presented patient as well. This step allowed us to perform asymmetric tailored craniotomies that avoided not only the frontal sinus on each side but also afforded best access corridors to both sides of this large OGM, without having to resort to either a large single piece bifrontal craniotomy that would have risked violating the PCG, or by relying on a unilateral approach that would have posed the same challenge, when pursuing tumor resection across the midline at the base of the anterior cranial fossa.

This is, to our knowledge, the first technical report of surgical resection of OGM in the setting of PCG, where the preoperative detection of this variant led to alteration of the surgical plan in favor of an individually tailored operative approach. While our preferred method for resecting OGMs remains a unilateral pterional craniotomy to limit iatrogenic morbidity, we decided here to employ a bilateral frontal approach without violation of midline structures with a favorable result. 

## Conclusions

In the preoperative work-up of OGMs, particular attention should be placed on detecting anatomical variations of the paranasal sinuses like PCG. Timely detection of this anatomic feature can prompt the surgeon to opt for an alternative operative trajectory that avoids violation of the aerated skull base and the associated increased risks of a hard-to-fix anterior skull base CSF leak and potential subsequent meningitis. We think that this anatomical scenario deserves further study in a well-chosen patient cohort of anterior cranial fossa tumors.
